# Electrophysiological Changes After Repeated Alcohol Withdrawal

**Published:** 1998

**Authors:** Larry P. Gonzalez

**Affiliations:** Larry P. Gonzalez, Ph.D., is an associate professor in the Department of Psychiatry and Behavioral Sciences, University of Oklahoma Health Sciences Center, Oklahoma City, Oklahoma

The chronic use of alcohol and other drugs leads to adaptive changes in the brain that serve to counteract the effects of the continuous presence of the “offending” drug. When the drug is subsequently withheld, these changes result in neurochemical imbalances that lead to various physiological and psychological responses. Withdrawal after extended alcohol exposure, for example, can result in mental confusion and motor disturbances ranging from tremors to seizures.

Studies in animal models consistently indicate that the manifestations of alcohol withdrawal (AW) can become progressively more severe after repeated withdrawal episodes. For example, some studies found that behavioral symptoms of AW (e.g., seizures) were more severe after two cycles of alcohol exposure and withdrawal than after one cycle (Branchey et al. 1971; Walker and Zornetzer 1974). This exacerbation of withdrawal symptoms is similar to the kindling phenomenon, which had first been observed in animal studies in the 1960’s. At that time, Goddard and colleagues (Goddard 1967; Goddard et al. 1969) analyzed the effects on behavior of brief, weak electrical stimuli administered through electrodes implanted at certain brain sites. Initially, these stimuli produced no observable behavioral response in the animals. After repeated, intermittent administration of the stimuli, however, the animals experienced convulsions. Later studies demonstrated that this kindling effect occurs not only following repeated electrical stimulation but also after repeated treatment with various chemical and sensory stimuli (Pinel 1980). Scientists currently believe that all of these stimuli induce a reorganization of the connections among nerve cells (i.e., neurons) in the brain, which results in kindling. Accordingly, kindling is an important example of the brain’s ability to adapt to changes in the body’s internal and external environments (Goddard and Douglas 1975; Morrell and de Toledo-Morrell 1986).

Since the 1970’s, numerous studies have provided evidence for kindling during AW (for a review, see Becker and Littleton 1996 and the article by Becker, pp. 25–33). Most of these studies, however, have focused on kindling of behavioral withdrawal symptoms. In contrast, few studies have investigated whether a kindling effect also occurs with respect to changes in the electrophysiological activity of the brain. This article reviews some of these studies and their implications for understanding the brain processes underlying AW. To date, these analyses have been limited to animal models.

## Electrophysiological Changes in Brain Activity After Repeated Alcohol Withdrawal

Walker and Zornetzer (1974) first suggested that the severity of withdrawal-related electrophysiological abnormalities in the central nervous system (CNS) might increase if the individual experienced multiple withdrawal episodes. A subsequent study supported the conclusion that repeated AW episodes result in a kindlinglike increase in electrophysiological changes in the CNS (Poldrugo and Snead 1984). Both studies compared the withdrawal severity (as measured by changes in electrophysiological activity in the CNS) in rodents after one period of alcohol exposure with that after two periods of alcohol exposure. This experimental design makes the results of these early studies difficult to interpret, however, because the animals were exposed not only to different numbers of withdrawal episodes but also to different total amounts of alcohol. Because the animals’ total alcohol exposure after two periods of alcohol exposure was twice that after one period, the rise in withdrawal severity could have resulted from the increase in total alcohol exposure rather than from the previous withdrawal experience.

**Figure 1:**
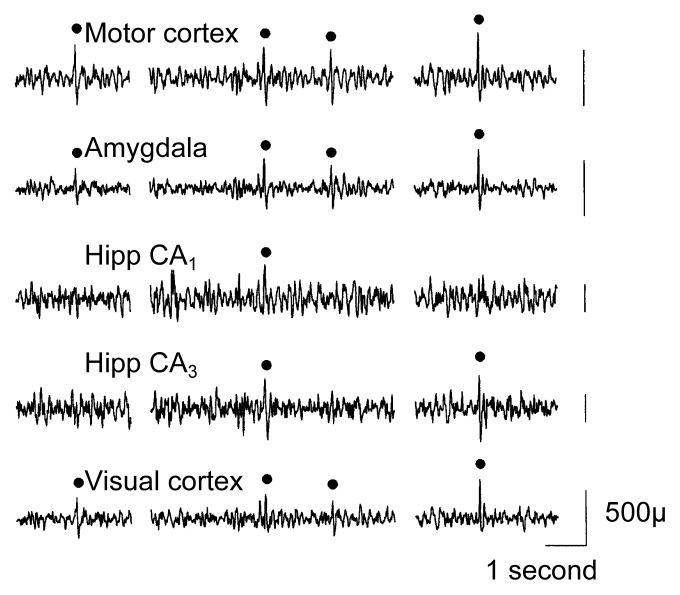
Sample electroencephalogram (EEG) recorded from a rat following alcohol withdrawal. Filled circles indicate computer-detected spikes and sharp waves, which are indicators of abnormal brain activity. Hipp = hippocampus. SOURCE: Adapted from Veatch and Gonzalez 1996.

Using a different experimental design to avoid these study limitations, Veatch and Gonzalez (1996) investigated the electrophysiological changes induced by chronic alcohol exposure and repeated withdrawal in rats. With this approach, both the total duration of alcohol exposure and the number of withdrawal episodes that the animals experienced were varied independently. The researchers surgically implanted electrodes into the animals’ brains, which allowed them to use electroencephalography (EEG) to record the animals’ brain wave activities in various cortical and subcortical brain structures.^1^ The animals were treated with several cycles of exposure to alcohol vapors (10 or 20 days per cycle), followed by 4-day withdrawal periods between alcohol exposure periods. Subsequently, the animals’ EEG activities were recorded intermittently for 3 days. The researchers then examined the EEG data for abnormalities in the brain waves, which appear as greater-than-normal spikes and sharp waves (SSW’s) on the EEG printout (see figure 1).

**Figure 2:**
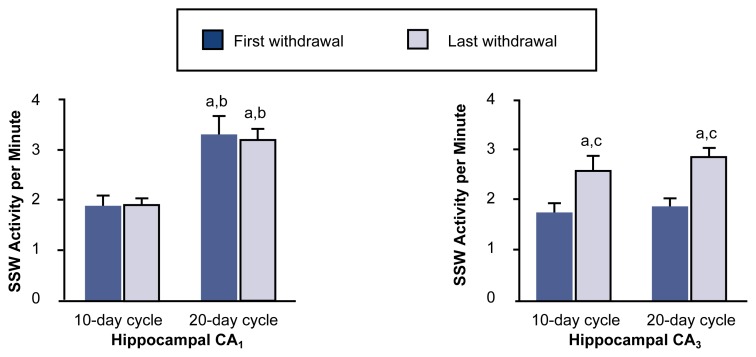
Effects of multiple cycles of alcohol exposure and withdrawal on abnormal brain waves (i.e., spike and sharp wave [SSW] activity) of rats. Rats were exposed to alcohol vapors either for two cycles of 10 days of alcohol exposure followed by 4 days of withdrawal or for three cycles of 20 days of exposure followed by 4 days of withdrawal. After the first and last withdrawal episode in each group, the SSW activity in two regions (CA_1_ and CA_3_) of the hippocampus^1^ was recorded by electroencephalogram (EEG) and averaged. SSW activity in the CA_1_ area was most affected by increases in total alcohol exposure, whereas activity in the CA_3_ area depended primarily on the number of withdrawal cycles. These results indicate that both total alcohol exposure and the number of withdrawal episodes influence electrophysiological activity during alcohol withdrawal. Moreover, different brain structures appear to be sensitive to different aspects of the alcohol exposure and withdrawal cycles. ^1^ The hippocampus is a region of the brain thought to play a role in learning and memory as well as in alcohol withdrawal seizures. ^a^Statistically significant (i.e., *p* < 0.05) compared with 10-day cycles, first withdrawal. ^b^Statistically significant (i.e., *p* < 0.05) compared with 10-day cycles, last withdrawal. ^c^Statistically significant (i.e., *p* < 0.05) compared with 20-day cycles, first withdrawal. SOURCE: Adapted from Veatch and Gonzalez 1996.

The study found that the extent of brain wave abnormalities depended on both the duration of the alcohol exposure and the number of withdrawal cycles, indicating the presence of a kindlinglike process. Moreover, different brain regions varied in their sensitivity to this kindlinglike effect. For example, increases in SSW activity were first observed in various areas of the hippocampus, whereas other subcortical and cortical sites exhibited increased SSW activity only after longer alcohol exposure or additional withdrawal episodes. Specific regions within the hippocampus (e.g., the CA_1_ and CA_3_ areas) also differed significantly from one another in their response to chronic alcohol exposure: Whereas SSW activity in the CA_1_ area was most affected by increases in the amount of total alcohol exposure, SSW activity in the CA_3_ area depended primarily on the number of withdrawal cycles (see figure 2). These results indicate that both total alcohol exposure and the number of withdrawal episodes influence electrophysiological activity during AW. Furthermore, different brain structures appear to be sensitive to different aspects of the alcohol exposure and withdrawal cycles.

## Relationship Between Electrical Kindling and Kindling During Withdrawal

Soon after electrical kindling of seizure activity and increased withdrawal severity after repeated withdrawal episodes were first observed, researchers suggested that both phenomena might be related. For example, Pinel and colleagues (1975) reported that animals previously exposed to electrical kindling stimulation exhibited exaggerated behavioral symptoms during a first withdrawal episode after chronic alcohol exposure. The investigators suggested that their findings indicated an association between exposure to convulsion-inducing treatments and AW severity. Subsequently, Ballenger and Post (1978) hypothesized that the abnormal neuronal activity occurring during AW may serve directly as a kindling stimulus, thereby increasing the incidence and severity of withdrawal symptoms during subsequent withdrawal episodes.

Studies such as the one by Veatch and Gonzalez (1996) described previously also suggest that the increased withdrawal severity after repeated withdrawal episodes may be caused by mechanisms similar to those underlying the increased seizure propensity observed after electrical kindling processes. To date, however, researchers have not yet demonstrated conclusively that both phenomena are the result of the same neuronal mechanisms. Such a demonstration will require additional studies of the neurophysiological changes that accompany both procedures.

Recent studies have shown, however, that chronic alcohol exposure, with or without intermittent withdrawal episodes, induces long-lasting changes in brain activity that influence subsequent electrical kindling processes. Veatch and Gonzalez (1997) exposed animals to alcohol vapors either for three cycles of 14 days each followed by 4 days of withdrawal or for one 42-day period. (Thus, both groups of animals were exposed to the same total amount of alcohol.) Two weeks after the final withdrawal episode, the researchers initiated electrical kindling by daily weak stimulation of the hippocampus both in the alcohol-treated animals and in control animals that had not been exposed to alcohol. The study found that although all animals exhibited the kindling phenomenon, both groups of alcohol-exposed animals required significantly more days of electrical stimulation to exhibit the same degree of convulsions compared with the control animals (i.e., the kindling response was delayed).

This finding was somewhat surprising because repeated withdrawal often is associated with increased susceptibility to seizures. Thus, if AW serves as a kindling stimulus, the number of electrical stimuli needed to complete the kindling process should be reduced after repeated AW. Several potential explanations exist for this apparent discrepancy. First, symptoms of increased excitability of the CNS, such as seizures, generally are observed only during the first few days of acute withdrawal. In the study by Veatch and Gonzalez (1997), however, kindling was not initiated until 2 weeks after AW. Second, studies of electrical kindling have indicated that kindling induced by stimulating one brain region may actually inhibit kindling processes induced by stimulation of a different brain region (Burchfiel and Applegate 1989; Duchowny and Burchfiel 1981). This finding suggests that kindling requires highly organized neuronal interactions and that a kindling process at one site or using one particular neuronal pathway may interfere with the organization of neuronal activity in other areas. AW affects many different brain regions. Consequently, this diffuse kindling stimulus may result in disorganization of neuronal activity at many brain sites, thereby inhibiting subsequent electrical kindling at those sites.

Although both continuous and repeated alcohol exposure delayed kindling development to a similar extent in the study by Veatch and Gonzalez (1997), certain differences existed in the responses of the two alcohol-exposed animal groups. For example, in animals that were continuously exposed to alcohol, the delay in kindling correlated significantly with the severity of acute withdrawal (i.e., the more severe the animal’s withdrawal symptoms were, the longer was the delay in electrical kindling). In contrast, no such correlation existed between withdrawal severity and delay of kindling in the animals that had experienced multiple withdrawal episodes. These observations suggest that after repeated cycles of alcohol exposure and withdrawal, sensitivity to kindling depends not only on the most recent withdrawal episode but also on the animals’ withdrawal history (which was the same for all animals in the group). Other studies have reported similar findings of alterations in electrical kindling following chronic alcohol exposure and withdrawal.

Similar to the changes in SSW activity detected after multiple AW episodes, the specific effects of previous withdrawal on sensitivity to electrical kindling depend on the specific brain regions analyzed. For example, McCown and Breese (1990) investigated the sensitivity of various brain areas to seizure induction by electrical kindling in animals that had undergone repeated AW episodes. Their study found that compared with control animals that had not undergone AW, seizures in the alcohol-exposed animals were induced more rapidly after stimulation of an area called the inferior colliculus, which is important in the processing of auditory information. Conversely, seizure induction was delayed after stimulation of the amygdala. Because the amygdala, like the hippocampus, contributes to the development and propagation of seizure activity in the CNS, these findings of delayed kindling of seizures suggest that repeated alcohol exposure and withdrawal may cause a long-lasting disruption in the functional organization of these brain structures.

## Summary

Studies of spontaneous and kindled electrophysiological brain activity following AW emphasize the important contribution of several variables to the severity of AW and to the associated long-lasting changes in brain function. Thus, withdrawal severity depends on the pattern of alcohol intake (e.g., withdrawal history) as well as on the total amount of alcohol exposure. Moreover, different brain regions respond differently to various patterns of alcohol exposure.

## Footnotes

1 The cortex is a thin layer of neurons (i.e., “gray matter”) on the surface of the brain. These neurons are involved in numerous functions, such as taste, speech, hearing, and abstract thinking. Subcortical structures are brain areas located below the cortex, such as the hippocampus and the amygdala. The hippocampus plays a role in memory formation. The amygdala is part of a group of brain structures that controls the expression of emotional behaviors.
